# Corvids optimize working memory by categorizing continuous stimuli

**DOI:** 10.1038/s42003-023-05442-5

**Published:** 2023-11-06

**Authors:** Aylin Apostel, Matthew Panichello, Timothy J. Buschman, Jonas Rose

**Affiliations:** 1 Neural Basis of Learning, Department of Psychology, Ruhr University Bochum, Bochum, Germany; 2 Department of Neurobiology, Stanford University, Stanford, CA USA; 3 Princeton Neuroscience Institute and Department of Psychology, Princeton University, Princeton, NJ USA

**Keywords:** Cognitive control, Computational neuroscience, Perception

## Abstract

Working memory (WM) is a crucial element of the higher cognition of primates and corvid songbirds. Despite its importance, WM has a severely limited capacity and is vulnerable to noise. In primates, attractor dynamics mitigate the effect of noise by discretizing continuous information. Yet, it remains unclear whether similar dynamics are seen in avian brains. Here, we show jackdaws (*Corvus monedula*) have similar behavioral biases as humans; memories are less precise and more biased as memory demands increase. Model-based analysis reveal discrete attractors are evenly spread across the stimulus space. Altogether, our comparative approach suggests attractor dynamics in primates and corvids mitigate the effect of noise by systematically drifting towards specific attractors. By demonstrating this effect in an evolutionary distant species, our results strengthen attractor dynamics as general, adaptive biological principle to efficiently use WM.

## Abstract (web summary)

Using a comparative approach to advance our understanding of working memory from an evolutionary perspective it is shown that corvid working memory has trade-offs between resolution and temporal stability, similar to what has been reported for primates.

## Introduction

Imagine you just had your morning coffee in your favorite café. Upon leaving you ask for your navy-blue umbrella that you had forgotten the day before. The waiter is proficient in remembering large orders in his day-to-day life. Yet, after taking the order from a different customer he brings a lavender blue umbrella that stood next to yours instead. Information held in memory tends to deteriorate over time, which results in a loss of precision and thus potentially explains why the waiter solely remembered ‘blue’ instead if the exact color shading.

The limitations of working memory (WM), the ability to maintain and manipulate information no longer present, have been extensively studied, often in the visual domain (see ref. ^1^ for a recent review on WM in primates). Commonly, studies ask ‘how many items’ subjects remember in order to assess WM capacity^2–5^, which has been shown to be strictly limited^2,4^. One cognitive strategy to reduce the demands of WM is to organize sensory input into discrete categories (i.e., remember ‘blue’ instead of royal or midnight blue). This categorization allows generalization across various sensory inputs that require the same behavioral response^6,7^, which is essential in our detailed and sensory rich environment^8,9^. However, grouping of information to reduce WM content also introduces additional sensory biases and inevitably reduces the precision of memory representations^10,11^ (e.g., leading to a confusion of umbrellas differing only in their exact shade of blue).

Over time, items in WM move away from their original state, becoming less precise the longer information is maintained^12–15^. Thus, errors accumulate over a prolonged memory period, which has been attributed to internal noise (random diffusion^12,16–18^). An additional drift of memory representations towards fewer, but relatively stable and diffusion-resistant states was proposed to counteract the impact of noise in primates^12,19^. These attractor dynamics draw memories towards a limited set of discrete representations, which mitigates diffusion but, by discretizing continuous information, also introduces systematic memory biases. Models that combined diffusion and drift towards attractors explain human and monkey behavior on a delayed estimation paradigm with colors^12^. Response distributions, precision, and bias indicated discrete attractor colors that were more precisely represented, less susceptible to noise (almost no drift), and dependent upon the statistics of the current environment—a biased target color distribution led to attractor states around more common colors^12^. Panichello et al.^12^ found that both humans and monkeys selected some hues more frequently than others, which was evident in the clustering of their response frequency; despite each color occurring as target equally often. This bias towards specific colors was also found in undelayed estimation (in humans), indicating potential origins in perception^19^ and a more general categorical representation of color^20,21^.

Categorical perception in the domain of color vision has been intensely studied^10,22–24^. Humans were shown to remember some colors better than others^19^ and to form discrete color categories that align with basic color terms, which are often comparable across different cultures and languages^25–27^. Besides color, representations of location and orientation have also been shown to present categorical biases^28,29^. Thus, a categorical representation of (continuous) sensory input might reflect a general strategy to cope with limited WM resources rather than a color specific effect. Therefore, exploring the precision and its interplay with capacity is an important approach to exploring the constraints of WM^19,30,31^. The former has frequently been examined with delayed estimation paradigms in which subjects must indicate a previously presented stimulus (color hue) on a circular (color) continuum^12,19,32–37^. Response precision and variability can be determined from the angular deviation between selected and previously presented stimulus (color hue) and investigated in conjunction with various modifications of WM demands.

Attractor dynamics in WM have been established for primates, yet it remains unclear if they constitute a general neurobiological principle, or if they are exclusive to mammals (or even only primates). To advance on this question, a distantly related species is required that is capable of producing comparable feats of WM, while having a differently organized brain. Previous studies demonstrated that WM capacity and neuronal computations were largely similar in primates and crows^2,38^, despite approximately 320 million years of separate evolution^39^. Corvids were shown to master WM tasks in a similar fashion to primates^2,38,40–42^ and can successfully be trained on complex behavioral tasks^43–46^. Furthermore, as in primates, visual perception is an important sensory modality in many bird species^47,48^. Hence, they represent an opportunity to examine if attractor dynamics can be generalized to a highly visual non-mammal with different forebrain architecture. Previous work demonstrated categorical perception in an avian species^23,24^, thus, a categorical bias towards more discrete memory representations based on attractor dynamics might be shared by diverse vertebrate groups, from birds to primates.

To investigate the dynamics of WM representations with distinct memory demands in birds, we adapted a delayed estimation paradigm previously used in primates. Overall, WM demand was manipulated by changes in delay duration and memory load to study the effect on performance and response accuracy. Our birds showed a decrease in performance and response accuracy with increasing WM demands. The responses of birds to specific target colors revealed discrete attractor dynamics (drift towards stable, noise resistant representations), which have been demonstrated in primates. Altogether, our comparative approach supports attractor dynamics as a general principle to mitigate the effect of noise on WM representations that are shared between mammals and birds.

## Results

Two jackdaws performed in this experiment, hereinafter referred to as ‘SPA’ and ‘ABR’. The birds completed a total of 119,790 (SPA) and 76,076 trials (ABR) across 276 and 271 sessions, respectively. On average, this corresponded to 434 ± 83.1 (SPA) and 279 ± 56.5 (ABR) completed trials per session (mean ± standard deviation). Overall, both birds achieved high performance across all experimental conditions.

### Birds memorized and selected target colors at high performance levels

**Fig. 1:**
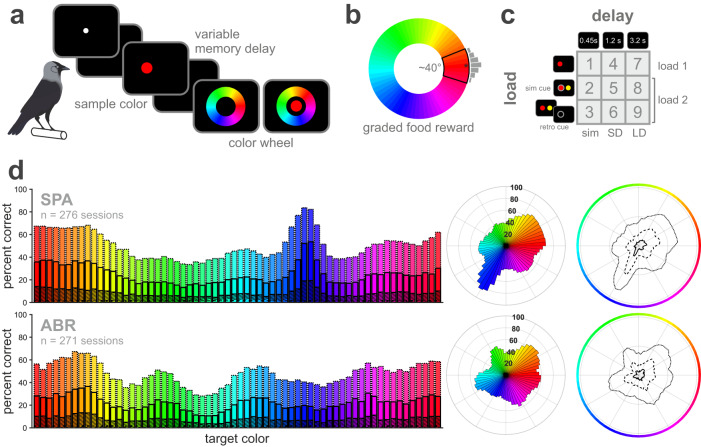
Delayed estimation paradigm with different delay durations and load conditions to manipulate WM demands revealed color-dependent performance differences in jackdaws. **a** Schematic view of the behavioral paradigm. The birds initiated a trial by pecking a white initialization stimulus. A specific sample color was shown that had to be remembered throughout a variable memory delay and selected from a choice stimulus in the form of a color wheel. **b** Full reward range included approximately 40° of the color wheel. The exact color (*corresponds to the largest gray bar) and three adjacent color patches (±3, clockwise (CW) and counterclockwise, CCW) were differentially rewarded (the more precise the higher the reward amount, level of gray bar symbolizes reward amount). **c** Overview of all nine different trial types. Each trial type represented a combination of three different delays (‘simultaneous’, short, and long delay) with three different load conditions (load 1, load 2 with simultaneous cue, and load 2 with retro cue). **d** Both birds showed performance differences depending on the target color. Performance per target color is shown for each accuracy level (target color-coded, accuracy level indicated by bar design/line style: ±3 = dotted; ±1 = solid/dashed; exact = hatched/solid). Chance level in the delayed estimation paradigm was at 11% correct (7 out of 64 colors, full reward range), 5% (3 out of 64 colors, inner reward range), or 1.6% (1 out of 64 colors, exact target color), respectively.

Two jackdaws were trained on a delayed estimation paradigm with 64 distinct sample colors. They obtained a graded food reward depending on their response accuracy. Different delay durations and load conditions were implemented to manipulate WM demands (Fig. 1a–c). Both birds responded significantly more often to colors within the full reward range than to colors outside of it (binomial test relative to chance level at 11% correct, *p* < 0.0001). Performance to correctly select a color from the color wheel differed significantly among the 64 target colors (all trial types pooled, Cochran’s Q test, SPA: ${{\rm X}}^{2}$ (63) = 7453.8, *p* < 0.0001, ABR: ${{\rm X}}^{2}$ (63) = 2762.3, *p* < 0.0001, Fig. 1d and Fig. S1a for more details). In accordance with the graded reward delivery, three different response accuracy levels could be differentiated. Overall, 50.9% (SPA) and 47.3% (ABR) of all responses lay within a range of seven colors (full reward range, ±3 colors around target color, dotted bars), 24.9% (SPA) and 22.2% (ABR) fell within a smaller range of three colors (inner reward range, ±1 color around target color, solid bars), and in 8.4% (SPA) and 7.7% (ABR) the birds reported the exact target color (hatched bars). The overall performance for each accuracy level is shown separately for each target color in Fig. 1d (polar plot, Fig. S1a for more detail). Generally, sample color affected the performance of both birds, who seemed to remember some colors better than others.

### Increased working memory demands reduced response accuracy and precision

**Fig. 2:**
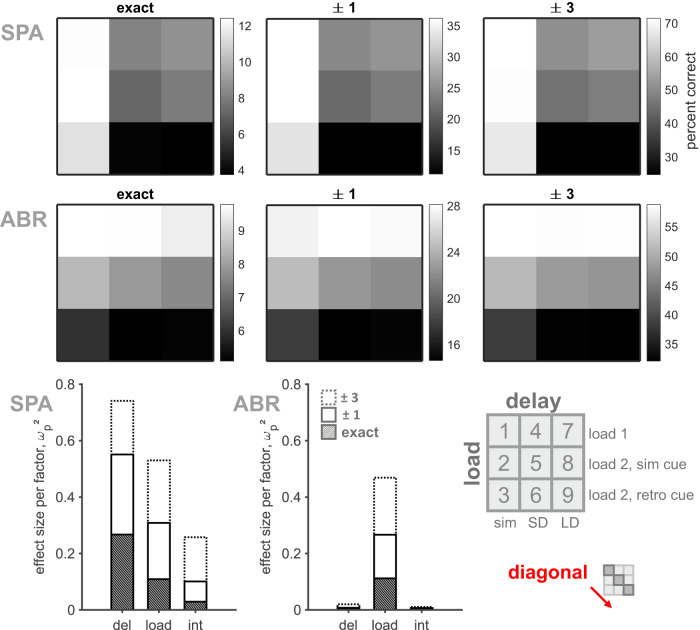
Performance varied with load and delay. Matrices visualize performance as a function of all combinations of load and delay for each accuracy level (data pooled over all sample colors, lighter fields indicate higher performance). Performance was shown to consistently decrease with increasing delay (SPA, top) and increasing load (ABR, bottom). Percent explained variance analyses confirmed delay (SPA), or load (ABR), respectively, as main factor to explain the largest proportion in performance variation (${\omega }_{p}^{2}$ as effect size measure, shown per factor and interaction in all three accuracy levels), which was also found in a non-parametric Friedman test (results see Table S2 and Fig. S2). Nonetheless, both birds showed a performance decrease with generally increasing memory demands along the diagonal.

Three different load conditions (load 1, load 2 with simultaneous and retro cue) were combined with three different delay durations (‘simultaneous’, SD, and LD) to create nine distinct trial types (Fig. 1c). Both task parameters, load and delay, and their interaction significantly affected the performance of both birds (two-way ANOVA, factors load, delay, and interaction, results see Table S1). However, the separate effects of both parameters on performance revealed individual differences, which were also apparent in the performance calculated per trial type (Fig. 2). SPA’s performance was mostly affected by delay, followed by load and the interaction of both factors. For ABR, most variation in performance could be explained by load. Performance generally decreased with the overall increase of WM demands in both birds. This increase in WM demands followed the pattern along the diagonal of the performance matrix: load 1, ‘simultaneous’ → load 2 + sim cue, SD → load 2 + retro cue, LD (referred to as ‘diagonal’, Fig. 2). Subsequent analysis focused on these trial types to visualize the effect of increased WM demands on various behavioral measures.

**Fig. 3:**
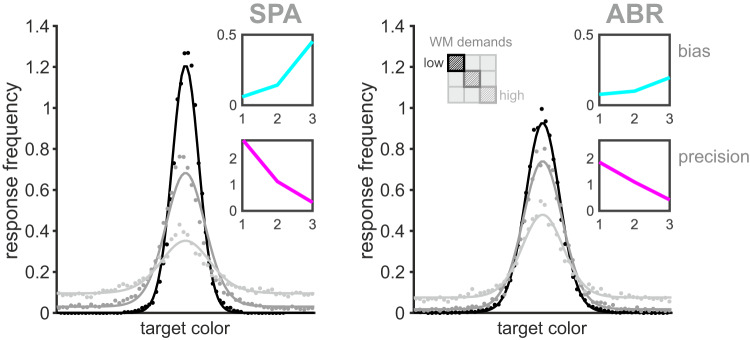
Error distributions reflected increase of WM demands. Responses became more variable with an increase in non-target and uniform responses due to increasing memory demands. Response behavior was characterized using the mixture model of ref. ^37^ (see methods, values in Table S3). Shown are trials along the diagonal (black: load1 + ‘simultaneous’; gray: load2 sim cue + SD; light gray: load2 retro cue + LD). Insets visualize mean absolute bias (cyan, top) and mean precision (magenta) along the diagonal (calculation based on ref. ^37^). Both birds showed a significant increase in absolute bias and significant decrease in response precision with increasing demands on WM.

We analyzed error distributions to investigate the effects of load and delay on response accuracy. Both birds demonstrated different response distributions and different proportions of target, non-target, and random responses depending on the trial parameters. We modeled the error distributions across the diagonal using a mixture model that estimates the response as a mixture of correct responses near the target color, random (uniform) guesses, and ‘swap errors’ when the animal responds with the color of the non-target distractor^37^. We found a decrease of the concentration parameter κ reflecting an increase in response variability for increasing memory demands. Further, both birds showed a decrease of target responses along with an increase in non-target and uniform responses (Table S3). Overall, response behavior was affected by task demands (load and delay). The general impact of increasing WM demands was visible in the error distributions of both birds along the diagonal (Fig. 3).

We further examined two additional, non-parametric measures of performance; the angular deviation of responses relative to the target color (bias, cyan curve Fig. 3) and the trial-to-trial variability in response error (inverse of the circular standard deviation, precision, magenta curve Fig. 3)^37^. Both measures were calculated individually per target color along the diagonal and then shown as average across all colors. For an increase of WM demands, both birds demonstrated a significant increase in mean absolute bias (i.e., they responded to colors increasingly distant to the target in either direction, SPA: ${\chi }^{2}=68.72,p < 0.0001$; ABR: ${\chi }^{2}=32.38,p < 0.0001$). At the same time, the response precision of both birds significantly decreased with increasing WM demands (SPA: ${\chi }^{2}=128,p < 0.0001$; ABR: ${\chi }^{2}=126.03,p < 0.0001$).

### General response behavior indicated a shift from continuous to categorical representation of color as memory demand increased

**Fig. 4:**
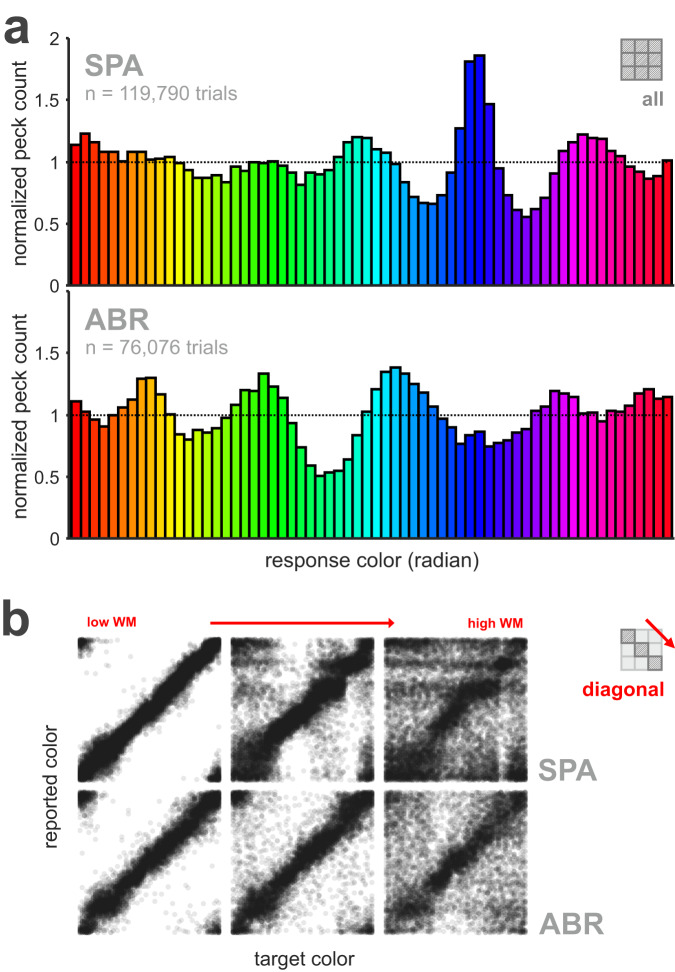
General response behavior revealed a categorical representation of color that was more pronounced for higher WM demands. **a** Normalized peck frequency per target color shows distinct peaks and troughs for both birds. Distribution of peck responses was clustered in comparison with an even distribution of presented target colors. Shown is normalized peck count, in which values > 1 indicate more and values < 1 less responses relative to the actual incidence. Data of all trials was pooled. The total number of trials per target color was 1872 ± 2.19 (SPA) and 1189 ± 5.94 (ABR, values represent mean ± SEM). **b** Response distributions reveal transition from continuous to categorical responding with increasing WM demands. Scatterplots visualize the distribution of reported color as a function of the actual target color along the diagonal per bird. In undelayed estimation, response distributions present a more or less straight line. With increasing WM demands, responses deviated stronger from the diagonal, presenting more categorical response behavior visible in emerging clusters, disrupted diagonal, or horizontal bands representing categorical guessing (SPA). Figure replicates analysis from refs. ^35,49^.

So far, we have shown color-dependent differences in performance and response accuracy. Categorical processing of color, in general, may also result in clustering of behavioral responses. To examine this, we calculated the overall response frequency of each color per target color, normalized by how frequently they were used as target colors (across all trials, Fig. 4a). In other words, values > 1 indicate more and values < 1 less responses relative to the actual incidence. Categorical responding was visible in the normalized peck frequency of both birds, who reported some colors more often (values > 1, distinct peaks) and others less often (values < 1, troughs). Figure 4a shows clustered, significantly non-uniform response distributions for both birds despite the uniform distribution of target colors (Hodges-Ajne test for non-uniformity of circular data, *p* < 0.0001 both birds, all trials pooled). In order to understand the effect of increased WM demands on the clustering strength, we visualized the distribution of reported colors as a function of target colors along the diagonal (as scatterplot following previous studies, e.g., refs. ^35,49^, Fig. 4b). A continuous, uniform distribution of responses along the color space should result in average responses near target values and thus be visible as a diagonal line. In contrast, more categorical responding would lead to a disrupted diagonal with staircase-like pattern and emerging clusters. In undelayed estimation with only one target color (Fig. 4b left), response distributions display a quite continuous straight line. Especially response distributions of SPA (top) suggest responses closely dispersed around each target color. With increasing memory demands, response distributions became more variable, showing stronger deviations from the diagonal. Overall, the proportion of random responses increased, showing responses more frequently further away from respective target color. Categorical responding became visible in horizontal bands indicative of categorical guessing, emerging response clusters, and an increasingly disconnected diagonal line (scatterplots for each separate combination of load and delay, see Fig. S3).

### Response behavior depended on target color

In addition, the accuracy and precision of responses depended on the target color (Fig. 1d polar line plot). This color dependency should also be reflected in the characteristics of response distributions for individual target colors with a non-uniform distribution of response parameters further supporting a categorical representation of colors. To investigate this, we visualized and modeled the error distributions separately for each target color (Fig. S4 and Table S4).

**Fig. 5:**
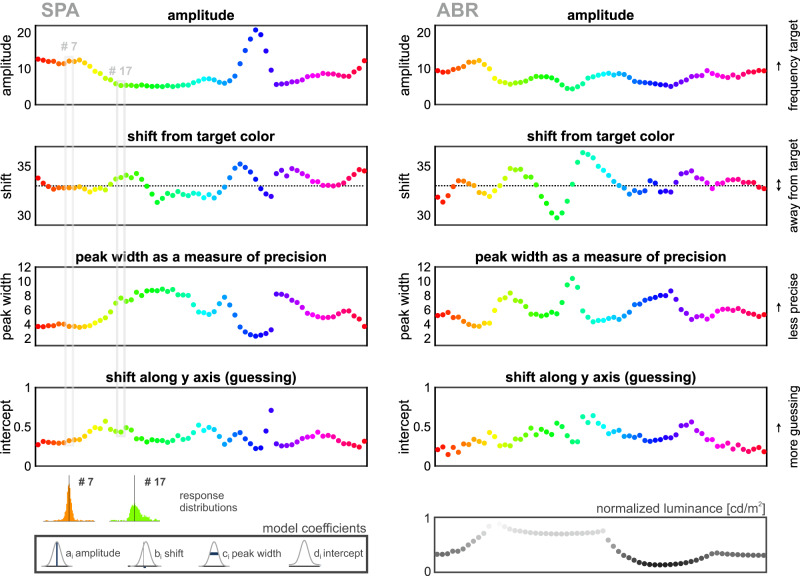
Response accuracy was color-dependent for both birds. Model coefficients visualize differences in the error distributions for specific target colors. Peak amplitude (a_i_), shift on the x-axis (b_i_), peak width (c_i_), and intercept (shift along *y*-axis, d_i_) varied with target color in the response behavior of both birds. Peaks in amplitude largely correspond to troughs in peak width as predicted by the attractor model. Gray boxes mark model coefficients of two exemplary response distributions of SPA (colors #7 and #17), shown below. Responses to the orange target color #7 reveal precise, accurate responding with minor bias and guessing. In contrast, responses to green color #17 were imprecise, more biased, and contained a pronounced uniform component. Normalized luminance values are shown in the same sequence per rendered target color. Although created to be iso-luminant, our rendered stimuli deviated from a consistent luminance value. Yet, differences in luminance between rendered stimuli did not match well with observed attractor colors.

Error distributions reflected the response accuracy, which showed a dependency on the specific target color. Responses to some colors were more accurate and precise compared to others. For example, response frequencies for red/orange target colors were characterized by a large amplitude (high response frequency for target color), negligible shift away from the target color (low bias), low peak width (high precision), and a low uniform component (e.g., color #7, Fig. 5). In contrast, responses to green hues generally had a smaller amplitude, larger shift away from the actual target color (stronger bias), broader peak width (lower precision), and a higher proportion of random responses (e.g., color #17, Fig. 5). We visualized response distributions per target color (histograms, Fig. S4) and characterized the error distributions per target color as average percentage of responses to individual colors by calculating separate model fits (see methods). To get an overview of the entire color range used, we compared the model coefficients obtained from each color fit to identify color-dependent differences in the response behavior (all model coefficients and adj. R^2^ per target color see Table S4). Individual Gaussian model fits of both birds revealed color-dependent differences in the response frequency (a_i_), CW and CCW deviations from the target color (b_i_), variances in peak widths (i.e., differences in precision, c_i_), and differences in the proportion of random responses (d_i_). Overall, the non-uniform parameter distributions revealed peaks and troughs that suggested a categorical representation of colors, consistent with the general response behavior described above.

Responses were not limited to the target color; they were influenced by the target color from the previous trial and by the simultaneously presented non-target color in load 2 trials. We compared the response bias in each trial with the deviation between current and previous target color and found that responses of both birds were attracted to the previous target color (Fig. S5a). This effect was also dependent on the absolute difference between both target colors with deviations around 76° (SPA) and 112° (ABR) causing the strongest serial bias effects (SPA: ~7.2°; ABR: ~5.8°). Note, that because colors were chosen randomly, these trial-by-trail biases cannot explain the systematic biases we observed for certain colors. We further visualized the general response behavior aligned to both target and nontarget color (Fig. S5b). A small peak in the response distributions relative to the nontarget color of both birds demonstrates an erroneous selection of the nontarget color in a small proportion of trials (often referred to as ‘swap errors’^50^). The frequency of swap errors in load 2 trials was 16.58% (SPA) and 19.31% (ABR).

### Birds categorized colors based on discrete attractors

**Fig. 6:**
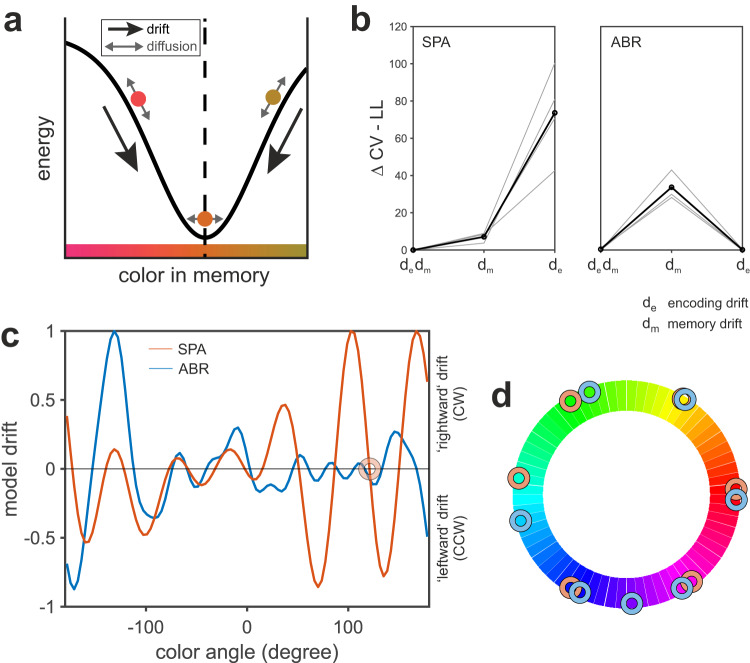
Drift-diffusion model of memory dynamics revealed discrete attractor colors that appeared similar in both birds. **a** Schematic of effect of drift and diffusion on memory. Local minima in a fit energy landscape across colors (i.e., attractors - dashed line) cause memories to drift over time (black arrow), introducing bias into reports. Noise causes memories to randomly diffuse (gray arrows). We assumed the strength of drift and diffusion differed between perception and memory delay. Fig. slightly adapted from ref. ^12^, published in Nature Communications under a Creative Commons license (http://creativecommons.org/licenses/by/4.0/). **b** Relative cross-validated negative log-likelihood of each model. Lower values indicate higher model likelihood. Gray traces: performance on each test fold. Black traces: average across folds. **c** Drift curves of SPA (orange) and ABR (blue). Orange circle shows an exemplary attractor of SPA identified as negative-slope zero-crossing (converging direction of drift). **d** Attractor colors were quite consistent between birds and evenly spread along the color wheel. In comparison to primates, birds had a higher number of attractor states (SPA: 6, ABR: 7, in comparison to monkeys (1 or 3), and humans (4)^12^).

The general response distribution and the normalized peck frequency illustrated categorical responding for both birds. In particular, an increase in clustering appeared in trials with increased memory demands (Fig. 4b). This categorical representation was further supported by color-dependent differences in response behavior for specific target colors (Fig. 5). To investigate the underlying memory dynamics of color representation, also in comparison to primates, we applied the drift-diffusion model of memory dynamics from Panichello et al.^12^ on the behavioral data of our birds. It describes how memories change over time based on two influences: color WM representations drift towards stable ‘attractor states’, which introduce biases into memory but are less sensitive to perturbation by random diffusion (Fig. 6a). The model assumes that both forces are present during both encoding and memory and their magnitude is fit independently for each load condition (see methods). Fitting this, and competing models, to our data allowed us to formally assess which classes of dynamics best explained behavior and illuminated the neural architectures that may underpin these dynamics^51,52^. Model comparisons indicated that the full drift-diffusion model provided a better account of the behavior of SPA than models in which drift does not act on memories during encoding or during the delays (Fig. 6b). In other words, for SPA, both the encoding and delay periods were best characterized by drift of memories towards discrete attractor states. These results are inconsistent with purely diffusive models of memory dynamics. For ABR, encoding drift was critical for explaining response behavior while including memory drift did not substantially influence model performance, as indicated by the small difference (0.27) in likelihood between the full and ‘no memory drift’ model (slightly favoring the latter). Inter-subject variability in cognitive models is common in human studies with large N and can be addressed via population summary statistics or hierarchical modeling^53^, and future work should take such an approach with a larger cohort of corvids to fully describe the distribution of dynamical schemes across the population. Nevertheless, these results provide evidence for discrete attractor dynamics during encoding and memory in corvids. Figure 6c illustrates the drift curves obtained for both birds. Discrete attractors are characterized by converging drift and are thus represented at negative slope zero-crossings (for example, attractor position indicated by the orange circle). Attractor colors were identified based on the respective drift functions and visualized within the color wheel (Fig. 6d and Fig. S6). Attractor colors were quite consistent between birds and evenly spaced along the color wheel. In comparison to primates, both birds had a higher number of discrete attractors (SPA: 6, ABR: 7, humans: 4, monkeys: 1 or 3^12^).

## Discussion

We trained two jackdaws on a delayed estimation paradigm with colors. Both birds showed accurate responding and high performance levels for all 64 target colors. An increase in WM demands (i.e., longer delay or higher memory load) resulted in a decrease of performance and response precision. The birds demonstrated individual differences in their response behavior, being more impaired by memory delay (SPA) or an increase in item load (ABR). Distinct clusters in the overall response distribution indicated a categorical representation of the color continuum. Some colors were reported with higher and others with lower frequency compared to their actual incidence as target color. Subsequent modeling of behavioral data confirmed specific colors as stable, noise resistant representations that were evenly spread along the color continuum and similar between birds.

We found color-dependent differences in performance already existed during perception, consistent with previous reports in human subjects^19,32^. Perceptual categorization was shown to occur spontaneously even in the absence of specific training^7,23,24,54^ and to emerge in various visual domains (color; refs. ^19,20,35,55^ location, orientation^56–60^). However, a general categorical perception of color is not easily distinguished from, for instance, the color rendering characteristics of the specific experimental setup. Birds are visual animals with accurate color vision. The avian visual system comprises four distinct single cone receptor types, some associated with colored oil droplets that further shape receptor peak sensitivities^47,61,62^. To our knowledge, spectral sensitivities of photoreceptors in jackdaws are still unknown, yet a tetrachromacy was demonstrated for most terrestrial birds^63^. Thus, the visible spectrum of birds stretches into the ultraviolet range containing wavelengths starting at 300 nm, ranging up to 700 nm^48,64^. Calibrating an experimental setup for avian (color) vision is technically challenging and would require both, bird adapted hardware and additional research to measure receptor pigments. However, to our knowledge, color vision details in corvids are still mostly lacking. Post-hoc measurements of dominant wavelengths of all rendered target colors used in our study revealed a non-linear relationship within our color range, although all 64 colors were equidistant in hue (Fig. S7a and Table S5). Therefore, based on the monitor display, some color hues could have been easier to discriminate than others, which presents a potential systematic confound in our study. Furthermore, despite being created with identical luminance, rendered colors also demonstrated unequal luminance values, which might have influenced the position of attractor colors. Yet, while some attractor colors seem to align with brighter or darker colors, luminance differences alone cannot explain all identified attractor colors nor minor differences between the two birds (Fig. S6). In general, physical stimulus features such as dominant wavelength or luminance cannot readily be used to make claims on actual perception^65,66^, which depends on the visual system (e.g., peak sensitivities of cone photoreceptors or their distribution). Thus, a perceptual (instead of a physical) reference metric would be necessary to evaluate jackdaw color vision. We cannot exclude that luminance besides color hue affected the attractor positions within the color wheel, which might also explain why we find relatively similar attractors in both birds. However, the memory related dynamics that were the central interest of this study cannot be explained by luminance differences alone (i.e., increase of response bias). Thus, the fact that we found changes in the color representation from encoding to memory suggests that our findings cannot be simply due to setup calibration and color rendering.

Instead of detailed color discrimination performance our focus was to examine WM dynamics in delayed estimation of birds. WM with color stimuli is often naively expected to be color independent and studies on WM capacity rarely consider differences in the memorability of certain color hues^33^. In contrast, and conforming with previous studies in primates, we found color-dependent differences in memories. Just as in humans, the birds’ responses to some colors were more dispersed and less precise compared to others^19^. The absence of response bias, narrow response distributions, and low guessing components characterized some colors as easier to remember than others. Clear peaks in the overall response frequency for specific target colors showed that both birds were biased in reporting certain colors more often than they were actually presented as target (Fig. 4a). This clustered, non-uniform distribution of responses mirrored previous findings in monkeys^12^ and human subjects^12,19^ and aligned with the specific attractor colors identified by the model. Although we cannot assess the precise position of identified attractors and determine whether they are perceptually salient due to specific color hue or physical luminance differences, we do show that there is an increased dependency on attractor states when WM demands are increased.

Consistent with findings in previous studies^19,55^, the delayed estimation performance of our birds decreased with increasing demands on WM – due to longer delays and higher memory load. We found that the decrease in performance of both birds was mostly explained by either delay duration (SPA) or memory load (ABR), suggesting slightly different individual strategies. For bird ABR, performance was markedly impaired in trials with multiple sample colors (even if the target color was presented again within the color wheel, i.e., trial types 2 and 3, Fig. 1c). Interference processes, like the concurrent presentation of multiple sample colors^32^, or the simultaneous presentation of the target color embedded within the cue or color wheel^19^, affected estimation performance in this animal. Besides color-dependent attractor dynamics, we found two additional interesting factors affecting the performance of our birds: the target color of a directly preceding trial, and a given trial’s non-target color. In our study, the responses of both birds were slightly shifted in the direction of the previous trial’s target color (Fig. S5a). Thus, sensory information from one trial influenced the behavioral performance on the next trial. The confounding effect of a stimulus presented in the preceding trial has been described, for instance, in the context of orientation^56,67,68^, and recently also in the color domain^69,70^—denoted as serial dependency (perception) or proactive interference (memory)^69,71,72^. Further, in some cases, the birds erroneously reported the non-target color instead, which has been characterized as a ‘swap’ or misbinding error that is often ascribed to noisy (memory) representations of the cued spatial location (small, but visible peak in the response distribution aligned on non-target color, Fig. S5b^37,50,73^,). Since non-targets are randomly distributed, such incorrect responses were intermixed with random guessing behavior (yet, a differentiation of specific error types was beyond the scope of our study). Both effects described above are likely confounded with attractor dynamics. However, these additional biases neither affect nor explain the main effects we found.

Generally, the observed decrease in delayed estimation performance reflected a random diffusion of WM representations due to increased memory demands and an increasing systematic drift of color memory (see model comparisons, Fig. 6b). This was reflected in the increase of the response bias for both birds (Fig. 3). The full drift-diffusion model provided the best account of the behavior observed in SPA, for which we found color representations to drift towards stable ‘attractor states’ during both the encoding and maintenance of color, which corresponds to our findings of categorical color representations on a perceptual and mnemonic level (Fig. S8a). For ABR, model results showed a strong influence of drift during encoding but no clear dependency on mnemonic drift. Yet, model-free analysis revealed an increase in response bias consistent with a stronger influence of attractor states for higher memory demands in this bird as well. Generally, the drift or bias towards attractor colors increased with memory demands (Fig. 3 and Fig. S8b), which is consistent with previous work in humans^55^ and an increase in random diffusion due to a prolonged retention period (i.e., longer memory delay) or competing signals of multiple memory items (i.e., higher memory load)^12,74^ as found in human subjects. An increase in noise or interference processes potentially increases uncertainty, which favors a categorical representation of color information to counteract or mitigate the effects of noise and to stabilize WM content^74,75^. Overall, we have found similarities in delayed estimation behavior between mammals (i.e., primates and humans) and birds (i.e., jackdaws). Based on the model drift functions we could identify six to seven discrete, evenly spaced attractor colors for both birds (SPA: 6, ABR: 7, Fig. 6d), which were consistent with peaks we found in the normalized response frequency per target color (Fig. 4a). The number of attractor-states we found in birds exceeded the numbers reported in monkeys (1 or 3) and humans (4) in a comparable study^12^, most likely due to differences in the experimental design. Attractor dynamics have been described as adaptive for the respective context and thus our narrower defined reward range (1/3, i.e., 40° instead of 120° as used in ref. ^12^) might have provoked a more thorough coverage of the full color range. However, it is important to keep in mind that a direct comparison of the number and location of attractor colors in humans, nonhuman primates, and birds remains challenging due to methodological differences (i.e., psychophysical stimulus properties). Generally, attractor states are not fixed to specific colors. Rather, they flexibly adapt to the stimulus set statistics. For example, if we would have selected our target colors from a more restricted color range, say only within the blue-green range, we would expect to find attractor colors adapted to this new circumstance (with sufficient experience). This would be in line with previous findings in humans^12^ and the view of attractor dynamics as general principle to reduce demands on WM irrespective of the particular memory content. Additionally, another interesting line of future research would be to analyze attractor dynamics using different sensory modalities, for example, auditory stimuli. Besides their excellent color vision, birds present a valuable comparative animal model for neuroscience in general. Investigating complex cognitive skills comparatively in avian species allows us to evaluate if behavioral traits and proposed models are truly dependent on specific neural architectures, such as the mammalian six-layered cortex, or if they constitute valid general principles of cognition^76^. Complex cognitive abilities on par with primates have been reported especially for members of the corvid songbird family. Corvids were shown to master complex cognitive skills such as tool use^77^, abstract categorization^45,78^, and working memory^2,42^. Several studies focusing on WM found no major differences between birds and mammals despite their long parallel evolution^42,79,80^. For instance, WM capacity and underlying neuronal computations were strikingly similar between crows and primates^2,38,40^. Due to their remarkable cognitive abilities and comparable WM dynamics, corvids are especially well suited as comparative animal model to probe recent models based on primate data. Following this, we found another striking similarity between corvids and primates in the general principles that support precise memory representations despite increased WM demands. Applying the model of ref. ^12^ to our data revealed comparable attractor dynamics in a non-mammalian species, suggesting comparable neuronal computations. Counteracting an increase of random noise with a tendency to categorically represent visual information appears to be an adaptive strategy for highly cognitive animal species that emerged from similar selection pressures.

Despite vastly different visual systems and brain organizations, corvids and primates show similar attractor dynamics, which can mitigate noise in visual working memory representations. Discrete attractors seem to be evolutionary conserved, not only across monkeys and humans, but also in corvids^12^. A categorical representation of color information in general with attractor colors as most representative examples seems to be an adaptive behavioral strategy to balance WM precision and limited capacity.

## Methods

### Subjects

Two hand-raised, experimentally naïve jackdaws (*Corvus monedula*) of undetermined sex (4 years of age) performed in this experiment (identified as ‘SPA’ and ‘ABR’). Both were randomly assigned from the colony kept in the lab (housing conditions were previously described in^81^). They were housed in a large indoor aviary at 20 to 22 °C room temperature in a social group under artificial daylight conditions (including 30-minute twilight phases, UV light, full color spectra, and high frequent illumination (5 kHz), ME International, Gallux). During the experimental procedures, the animals were held on a controlled food protocol with *ad libitum* access to water and grit (free feeding weight of both birds was 250 and 190 g, respectively). Special bird food pellets were used as reward during training (NutriBird F16, Versele Laga) and a mix of seeds, dried/fresh fruits, dried insects, mealworm larvae, and two bird foods (Beo-Weichfutter, Trocken-Weichfutter III Braun, Claus) supplemented with Korvimin (vitamin product, ZVT + Reptil) was given on days without training. We have complied with all relevant ethical regulations for animal testing. All experimental conduct was in agreement with the European Communities Council Directive for the care and use of animals for experimental purposes and approved by the local authorities (LANUV NRW).

### Experimental setup

All training and testing were conducted in a darkened operant conditioning chamber (80 × 54 × 56 cm; height × width × depth) equipped with an acoustic pulse touchscreen (22”, ELO 2200 L APR, Elo Touch Solutions Inc., CA) and an automated pellet feeder (https://www.ngl.psy.ruhr-uni-bochum.de/ngl/shareware/pellet-feeder.html.en). The chamber was insulated with acoustic foam to reduce background noise, which was additionally masked by a ventilation fan. The birds were seated on a wooden perch, approximately 10.5 cm from the screen. All experimental procedures were controlled from a computer running custom MATLAB code using the Psychophysics^82^ and Biopsychology toolboxes^83^.

### Behavioral task

Two jackdaws were trained on a delayed estimation paradigm adjusted for birds based on the paradigm of ref. ^12^ (Fig. 1a). The birds had to select a previously presented sample color from a continuous choice stimulus (‘color wheel’, 8.5 cm in diameter, consisting of 64 uniformly distributed colors, Fig. 1b). Colors were specified in the HSV color space with equidistant values for hue and consistent, maximum values for saturation and value, i.e., brightness. Sample colors were randomly drawn from the 64 colors and presented at two possible spatial locations (horizontally adjacent, Fig. 1c). Sample colors were presented as colored dots, 2.5 cm in diameter. A spectroradiometer (Konica Minolta Chroma Meter CS-150) was used to measure the rendered dominant wavelength and luminance of each sample color (Table S5).

### Trial structure

The birds had to initiate each trial by pecking a white initialization stimulus. After a short 500 ms delay, one or two sample colors were presented for 800 ms (target and non-target color). Following a variable delay period, the color wheel choice stimulus was presented randomly rotated to prevent spatial biases (Fig. 1a). The birds had to selectively peck the previously presented target color to obtain a food reward (target color in load 2 trials was indicated by a gray circle). The reward amount was dependent on the deviation between target and reported color. A peck to the exact target color produced the full reward amount (3 pellets), which was gradually reduced for a response within the inner (±1 color, 2 pellets) and outer (±3 colors, 1 pellet) reward range. The full reward range contained ~ 40° or seven distinct colors of the color wheel, including the three adjacent colors both clockwise (CW) and counterclockwise (CCW) from the target color (Fig. 1b). Incorrect or imprecise responses deviating by more than three colors from the target color led to a short screen flash (error signal) followed by a 10 s time-out. A 10 s inter-trial interval was used.

### Trial types

Both memory load and delay duration were manipulated. The full paradigm consisted of nine different trial types (Fig. 1c), representing unique combinations of three different load conditions and three different delay durations. Trials with one or two sample colors were used. In load 1 trials, only one sample color was presented as target color. In load 2 trials, a gray circle served as cue to indicate which sample color had to be reported. It was present for 0.2 s, either as simultaneous cue together with both sample colors or at the end of the delay period as retro cue. The three different delay durations used were 0.45 s (‘simultaneous’), 1.2 s (SD), or 3.2 s (LD), kept constant for all load conditions (i.e., whether a retro cue was presented in a given trial or not). In trials with the shortest delay, the target color was shown again centered inside the color wheel stimulus during the choice period, denoted as ‘simultaneous’ delay (representing an approximation of undelayed estimation, Fig. 1a). The order of trial types and target positions (‘left’ vs. ’right’) were pseudorandomized. Both sample colors per trial (target and non-target in load 2 trials) were completely randomly selected from the 64 distinct colors used in this study.

### Data collection and analysis

Data analysis was performed using MATLAB (MathWorks, R2020b). For each peck response, the respective color identity was calculated instantaneously based on the peck coordinates received from the touchscreen, the structure, and rotation of the color wheel stimulus. The deviation between this ‘reported color’ and the actual ‘target color’ determined the reward amount that was given per trial. The exact peck coordinates within the color wheel choice stimulus were later used to calculate the angular deviation relative to one set reference color (see Fig. S7b for an exemplary distribution of pecks during one session). All completed trials, in which the birds made a response to the color wheel (either rewarded or too imprecise and thus punished) were used for data analysis. Behavioral performance, response accuracy and error distributions per target color were calculated using the ‘reported color’ ID (colors labeled with 1 to 64). The behavioral performance quantifies the ratio of correct responses calculated as the number of correct trials divided by the total number of trials. To distinguish different levels of response accuracy, three different reward ranges were used (i.e., response to the exact target color, inner, and full reward range). Analysis of response distributions, peck frequency, response precision, bias, and modeling (mixture model, drift-diffusion model) were based on the angular deviation of peck response or sample colors relative to the reference color (values in radian, in the range of -π to π). Figures were generated with MATLAB and CorelDRAW 2018.

### Statistics and reproducibility

Statistical tests were calculated in MATLAB. In addition, the CircStat Toolbox (Hodges-Ajne test)^84^, and Cochran’s *Q* Test^85^ were used for data analysis and code from Ikuma^86^ was used for visualization (hatched bars, Fig. 1d). Response bias (circular mean error) and precision (inverse of circular standard deviation, corrected for chance) were calculated separately for each target color using code by ref. ^37^ (http://bayslab.com). We calculated a binomial test to analyze if the performance was significantly higher than chance level at 11% correct (7 out of 64 colors, full reward range, see results). We based this analysis on a theoretical value combining the lowest performance and lowest trial number to utilize the most conservative data-driven approach. We further analyzed if the performance differed significantly between target colors performing a Cochran’s *Q* test^85^ (see results), which uses binary input data (i.e., 0 or 1, specifying performance per trial) to calculate if different data groups have the same number of successes and failures. To analyze the separate effects of load and delay we used a two-way ANOVA with factors ‘load’, ‘delay’, and their interaction (see Table S1). We used partial ${\omega }^{2}$ as effect size measure, interpreted as percentage of explained variance (PEV, see Fig. 2). We have performed additional non-parametric Friedman tests and calculated Kendall’s W as effect size estimate for each factor (see Supplementary Table S2 and Fig. S2). Differences in the average absolute bias and response precision were analyzed with a Friedman’s test (see results) and a Hodges-Ajne test^84^ was used to test for non-uniformity of multimodal distributions of peck frequencies (see results). The error distributions as a function of overall memory demands were modeled using the mixture model of ref. ^37^ (see Fig. 3 and Table S3). The proportion of trials in which the nontarget color was reported instead of the actual target color (‘swap error’) was calculated using code from ref. ^50^ (see results). Responses to individual target colors were characterized with a Gaussian fit as close approximation (see Fig. S4 and Table S4) and the drift-diffusion model by ref. ^12^ was used to investigate the dynamics of color representations in working memory (see results, Fig. 6). We have used an alpha of 0.05 when making any statement on significance. Statistical details are reported within the results section or additional tables within the supplementary material as indicated above.

### Modeling of error distributions

Error distributions as a function of distinct memory demands were analyzed using the mixture model of ref. ^37^ (www.paulbays.com). For this, we used the angular deviations of peck responses and sample colors focusing on distinct trial types (values in radian, within the range -π ≤ x < π). This probabilistic mixture model estimates the proportion of three different components that are contained in the overall response distribution: target responses (correct report of cued target), non-target responses (erroneous report of un-cued non-target), and uniform responses (random, unrelated to target or non-target). It is based on an EM (expectation–maximization) algorithm and returns log likelihood of the fitted model (LL) and four output values describing the maximum likelihood estimates of model parameters (B = [κ, pT, pN, pU], with κ representing the concentration parameter of the von Mises distribution, and [pT, pN, pU] indicating the estimated probability of target, non-target, and uniform responses).

Error distributions per target color were modeled using a Gaussian approximation to the mixture model. This approach was pursued to obtain a detailed characterization of the response behavior per color. For each target color and all completed trials, the percentage of responses to each of the 64 colors within the color wheel was calculated and averaged across sessions (using the reported color ID). In order to visualize the response distributions relative to the centered target color, the data was shifted accordingly (which resulted in all target colors being present at position 33). We then fitted a Gaussian distribution to each data set using the following equation (nonlinear least squares as fitting method, Eq. 1):

1 $$y=\mathop{\sum }\limits_{i=1}^{n}{a}_{i}e{\left(-\frac{(x-{b}_{i})}{{c}_{i}}\right)}^{2}+{d}_{i}$$

The four model coefficients amplitude (a_i_), shift from the target color (b_i_), peak width (c_i_), and shift along the *y*-axis (intercept as measure of random responses, d_i_) were used to characterize the response behavior per target color. As initial values for coefficients we used a_i_ = 1, b_i_ = 33 (position of shifted target color as expected peak position), c_i_ = 1, and d_i_ = average number of responses to the farthermost 30 colors (colors 1–15 and 50–64). The adjusted R² served as measure of the goodness-of-fit. This was done for each target color separately (i.e., from i = 1 to *n* = 64).

### Drift-diffusion model

To investigate the dynamics of color representations in working memory, we modeled the evolution of memories over time as a drift-diffusion process^12^ (https://github.com/buschman-lab/WorkingMemoryDynamics). In brief, this model describes how memories change over time based on two influences. First, systematic biases may cause memories to drift towards stable attractor states over time. Second, memories may be perturbed by random noise. The model assumes that these forces may act instantaneously during encoding or accumulate over time in memory. Drift and diffusion represent an influence on memory representations during encoding and memory delay, and these values are fit independently for each load condition. Fitting this and competing models to our data allows us to formally assess which class of dynamics best explains behavior and illuminates the neural architectures that may underpin these dynamics^51,52^.

More specifically, we assume that memories evolve according to a stochastic ordinary differential equation that captures the influence of both systematic drift and random noise (diffusion, Eq. 2):

2 $$d\theta ={\beta }_{L}G\left(\theta \right){dt}+{\sigma }_{L}{dW}$$

This equation describes the time evolution of a color memory $\theta$ (a circular variable corresponding to an angle in the circular color space) under the influence of some deterministic dynamics defined by *G* (the drift) as well as an additive white noise process *W* with variance ${\sigma }^{2}$. ${{{{{{\rm{\beta }}}}}}}_{L}$ sets the gain of the drift. To account for the fact that memory load may influence these dynamics, we fit a separate $\beta$ and $\sigma$ for each load condition. We excluded simultaneous cue trials from this analysis because the load on these trials was ambiguous. $G\left(\theta \right)$ was a nonlinear function fit to each bird during model estimation using a linear combination of von mises derivatives separated by 1 standard deviation (fixed to $2\pi /12$) on the interval $({{{{\mathrm{0,2}}}}}\pi )$.

To fit this model to our behavioral data, we needed to describe the time evolution of $\theta$ probabilistically. So, we rewrote Eq. 2 as a Fokker-Planck equation, a partial differential equation that tracks the probability density function of $\theta$ over time: $p(\theta ,t)$ (see ref. ^12^ for full details). To dissociate load-driven changes in the dynamics of memory and encoding, we allowed the state of the memory to vary at the start of the delay. Specifically, when modeling each trial, we allowed Eq. 2 to evolve for a 1-second encoding period with an encoding and load-specific $\beta$ and $\sigma$. Dynamics were then dictated by Eq. 2 according to a delay- and load-specific $\beta$ and $\sigma$ for the duration of the memory delay. To account for so-called swap errors and guessing, the final response distribution was computed as a mixture of the target memory distribution, the non-target memory distribution, and a uniform component^12^.

We identified the maximum likelihood estimate (joint likelihood across trials) of the model parameters using gradient descent. For computational tractability, a random subsample of 3000 trials from each load-delay condition was used for fitting. To determine if drift during encoding and/or the memory delay were necessary to explain behavior, we also computed the log-likelihood of the best-fitting models when $\beta$ was set to zero during encoding (no encoding drift) and during the delay (no memory drift). To perform model comparison, we randomly partitioned each animal’s data into 4 folds, training each model on 3 folds and computing the negative log-likelihood of the model on the held-out fold. The differences in negative log likelihood (and the average across folds) are displayed in Fig. 6b.

### Reporting summary

Further information on research design is available in the Nature Portfolio Reporting Summary linked to this article.

### Supplementary information

Supplementary Information

Description of Additional Supplementary Files

Supplementary Data 1

Reporting Summary

## Data Availability

The data that support the findings of this study are available from the corresponding author upon reasonable request. All numerical source data for graphs and charts reported in this article is available under the following 10.5281/zenodo.8385549^87^ or from Supplementary Data 1.

## Code availability

The code that supports the findings of this study is available from the corresponding author upon reasonable request.
