# Supplementary material for: Corvids optimize working memory by categorizing continuous stimuli
Source: Commun Biol. 2023 Nov 6;6:1122. doi: 10.1038/s42003-023-05442-5 (PMC10628182; doi:10.1038/s42003-023-05442-5)
Supplement: Supplementary file 1 — Supplementary Information [file 42003_2023_5442_MOESM1_ESM.pdf]

Supplementary information,

Corvids optimize working memory by categorizing continuous stimuli

Aylin Apostel, Matthew Panichello, Timothy J. Buschman, Jonas Rose

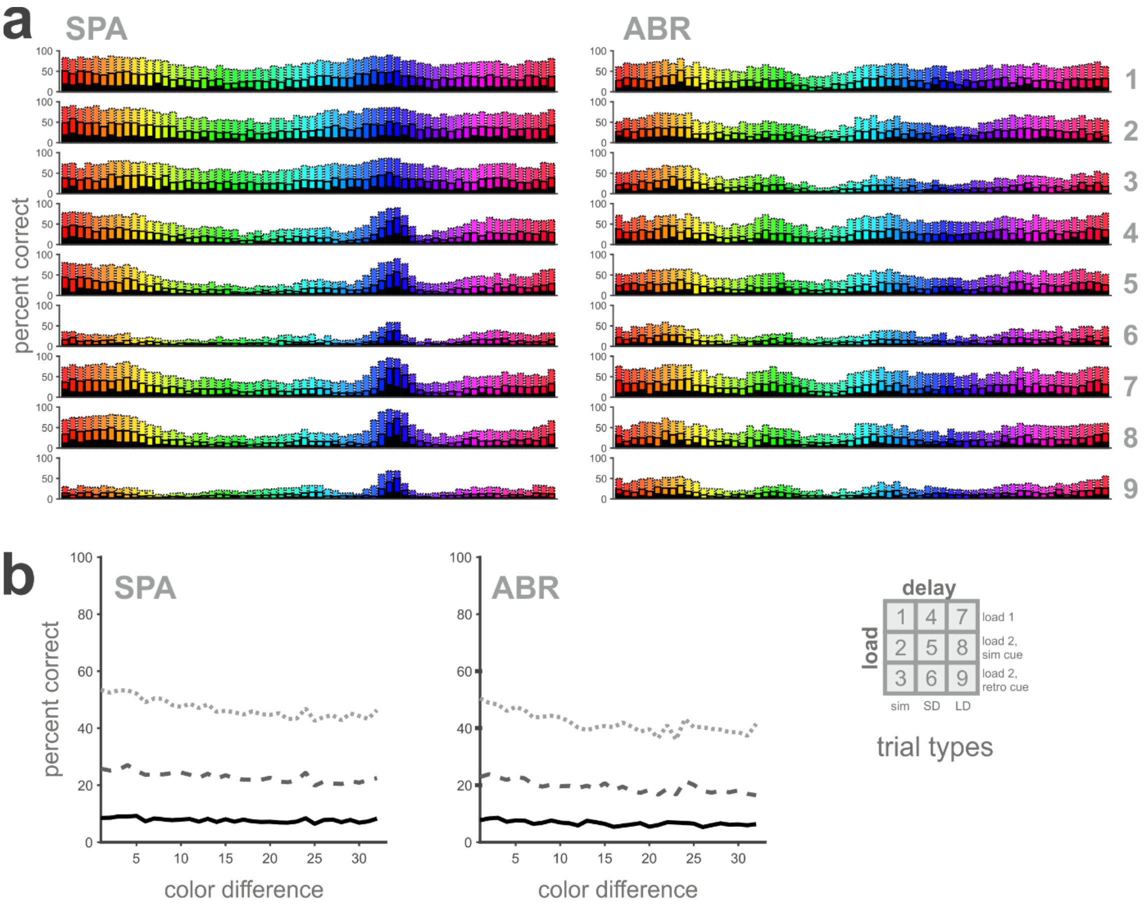

Fig. S1: Performance differed per target color and memory demands but was relatively independent of sample color difference. a Performance decreased with increasing memory demands. Performance per target color is shown per delay – load combination and for each accuracy level (target color-coded, accuracy level indicated by bar design: ±3 = dotted; ±1 = solid; exact = solid black). Chance level in the delayed estimation paradigm was at 11 % correct (7 out of 64 colors, full reward range), 5 % (3 out of 64 colors, inner reward range), or 1.6 % (1 out of 64 colors, exact target color), respectively. A decrease in performance was obvious for an increase in delay duration and memory load (parameter per trial type, see legend). For more detailed explanation of trial types 1 to 9 see Fig. 1 in main paper. **b** Performance was mostly independent of sample color difference in both birds. Performance is shown separately for all three accuracy levels (indicated via line style, ±3 = dotted; ±1 = dashed; exact = solid). Sample color difference was calculated irrespective of CW or CCW deviation (maximum difference was 32 indicating opposite positions within the color wheel).

Table S1: Effect of increased memory load or delay duration on behavioral performance. Separate effects of load and delay on behavioral performance were analyzed conducting a 2-way ANOVA with factors load, delay, and interaction, calculated for each bird and all three accuracy levels.

	**exact**			**± 1**			**± 3**		
	load	del	load*del	load	del	load*del	load	del	load*del
**SPA**	F(2,2475) = 152.7609; p < .0001	F(2,2475) = 453.7493; p < .0001	F(4,2475) = 19.4896; p < .0001	F(2,2475) = 554.8243; p < .0001	F(2,2475) = 1525.1; p < .0001	F(4,2475) = 70.4948; p < .0001	F(2,2475) = 1401.2; p < .0001	F(2,2475) = 3559.4; p < .0001	F(4,2475) = 216.4809; p < .0001
**ABR**	F(2,2429) = 154.2925; p < .0001	F(2,2429) = 4.1504; p = .0159	F(4,2429) = 0.6370; p = 0.6361 n.s.	F(2,2429) = 443.8946; p < .0001	F(2,2429) = 10.1649; p < .0001	F(4,2429) = 4.9817; p = .0005	F(2,2429) = 1077.0; p < .0001	F(2,2429) = 25.6002; p < .0001	F(4,2429) = 7.3462; p < .0001

Table S2: Effect of increased memory load or delay duration on behavioral performance. Separate effects of load and delay on behavioral performance were analyzed conducting a non-parametric Friedman test for factors load and delay, calculated for each bird and all three accuracy levels. For ABR, one session was removed from this analysis due to the lack of trials from one trial type. Kendall's W was calculated as effect size estimate for each factor (Fig. S3), which follow the same overall pattern as the ω_p^2 (Fig. 2).

	**exact**			**± 1**			**± 3**	
	load	del		load	del		load	del
**SPA**	X^2(2,2475) = 324.6287; p < .0001	X^2(2,2475) = 617.4146; p < .0001		X^2(2,2475) = 708.8032; p < .0001	X^2(2,2475) = 1187.8; p < .0001		X^2(2,2475) = 942.0026; p < .0001	X^2(2,2475) = 1502.6; p < .0001
**ABR**	X^2(2,2421) = 278.5748; p < .0001	X^2(2,2421) = 8.6136; p = .0135		X^2(2,2421) = 708.4707; p < .0001	X^2(2,2421) = 23.9889; p < .0001		X^2(2,2421) = 1227.9; p < .0001	X^2(2,2421) = 57.3443; p < .0001

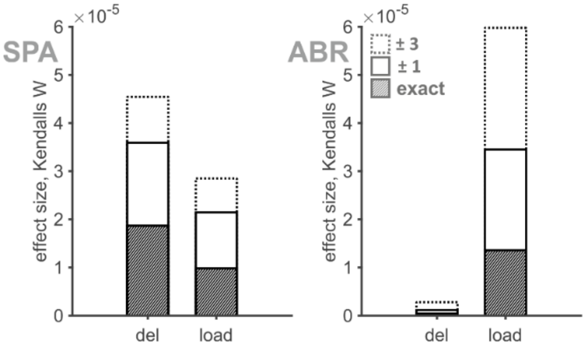

Fig. S2: Effect size estimate for Friedman test. Kendall's W reveal the same pattern of effect size estimates for factors delay and load compared to the ω_p^2 reported in the main paper (Fig. 2) (SPA left, ABR right).

Table S3: Overview of model estimates obtained from fitting the mixture model to trials with different WM demands. SPA (top), ABR (bottom). Error distributions were modeled separately across the diagonal (i.e., trial types 1, 5, and 9), visualized in Fig 3. Stated are log likelihood of the fitted model (LL) and four maximum likelihood estimates of the parameters of the mixture model: κ concentration parameter of the von Mises distribution (i.e., reflects response variability), and the estimated probability of target (pT), nontarget (pN), and uniform (pU) responses. Error distributions were modeled using code from Bays et al.[1].

SPA		LL	maximum likelihood parameters			
			K	pT	pN	pU
type	1	-4612	9.5129	0.9953	0.0000	0.0047
	5	-15225	4.4134	0.8043	0.1073	0.0884
	9	-21542	2.9194	0.3997	0.2893	0.3110

ABR		LL	maximum likelihood parameters			
			K	pT	pN	pU
type	1	-5355	5.8085	0.9856	0.0000	0.0144
	5	-8550	4.4433	0.8879	0.0653	0.0468
	9	-11769	4.0283	0.5238	0.3325	0.1437

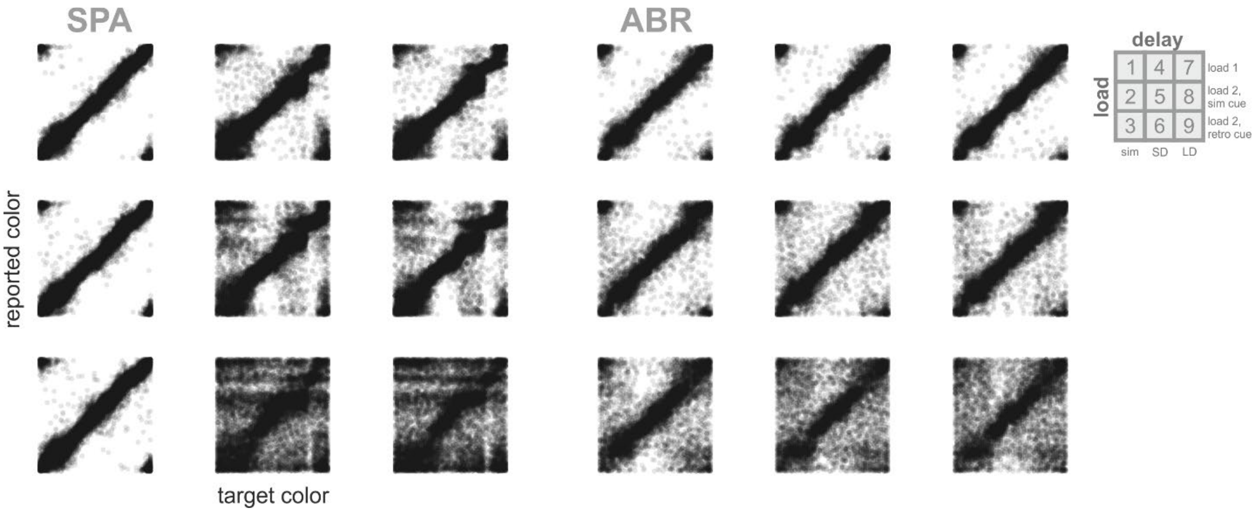

Fig. S3: Response distributions reveal continuous and categorical responding. Shown scatter plots visualize the distribution of the reported color as a function of the actual target color per trial type and bird (SPA left, ABR right). In undelayed estimation, response distributions present a roughly straight line. Distinct clusters emerge as the demands on WM increase, some showing sudden changes suggestive of category boundaries (along load and delay). Fig replicates analysis from[2,3].

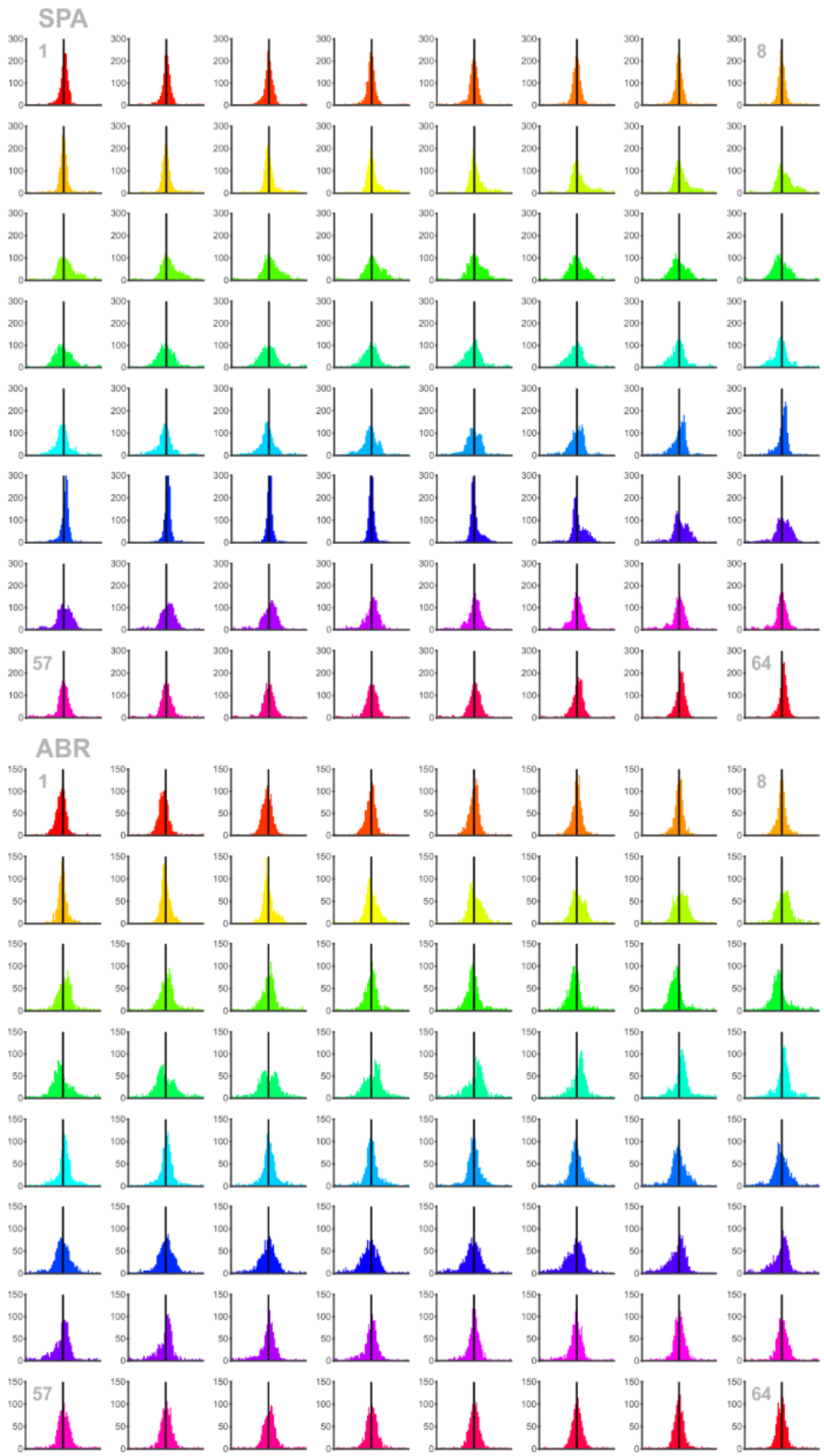

Fig S4: Individual histograms of response distributions per target color. SPA (top) and ABR (bottom). Shown are all target colors 1 to 64 (from left to right, top to bottom).

Table S4: Overview of model coefficients obtained from Gaussian fits to individual target colors. The percentage of responses to each color was calculated per target color and averaged across sessions. Before calculating the model fits, this data was shifted to center each target color at position 33. SPA (left), ABR (right). Stated are the adjusted R^2 as goodness-of-fit measure and the four model coefficients amplitude (a_i), shift (b_i), peak width (c_i), and intercept (d_i).

SPA	GOF	coefficients			
color	adj. R^2	a_i	b_i	c_i	d_i
1	0.9824	12.6556	33.7924	3.6806	0.2725
2	0.9866	12.3647	33.2731	3.6476	0.3134
3	0.9765	12.0061	32.9358	3.7891	0.3026
4	0.9843	12.0574	32.6682	3.7893	0.2972
5	0.9917	11.3697	32.8107	4.0348	0.2920
6	0.9944	11.4053	32.7833	4.0045	0.2976
7	0.9875	12.1148	32.8073	3.693	0.3234
8	0.9877	11.9969	32.7861	3.6957	0.3346
9	0.9815	12.4097	32.9047	3.5651	0.3373
10	0.9931	11.6248	32.6862	3.6724	0.3802
11	0.9840	10.6689	32.3802	3.7915	0.4422
12	0.9663	9.2685	32.6373	4.1747	0.4909
13	0.9445	8.6573	32.7689	4.5258	0.4774
14	0.9451	7.3103	32.5901	4.8963	0.5712
15	0.9486	6.9049	33.164	5.7258	0.4676
16	0.9099	5.8209	33.6962	6.9442	0.4430
17	0.9256	5.3220	33.8469	7.6585	0.4337
18	0.9208	5.3779	34.0884	7.2056	0.4893
19	0.9318	5.3727	33.8299	7.4130	0.4595
20	0.9361	5.1781	34.2595	8.4718	0.3476
21	0.9331	5.4627	33.6675	8.0154	0.3499
22	0.9479	5.2300	32.9677	8.6621	0.3079
23	0.9306	5.0713	32.1631	8.7387	0.3352
24	0.9348	5.1463	31.3176	8.6851	0.3246
25	0.9612	5.0141	31.7265	8.9093	0.3253
26	0.9643	5.2939	32.2282	8.5895	0.3032
27	0.9662	5.0379	32.0249	8.8568	0.3268
28	0.9629	5.2272	32.423	8.1175	0.3874
29	0.9458	5.4616	32.1268	8.0889	0.339
30	0.9484	5.4361	32.0051	8.0293	0.3537
31	0.9447	6.104	32.2549	6.7237	0.4259
32	0.9322	6.5863	32.1865	5.7046	0.5219
33	0.9617	7.1385	31.7892	5.6109	0.4532
34	0.9637	7.1904	32.108	5.3724	0.4927
35	0.9655	6.8868	31.7702	5.7535	0.4652
36	0.9471	6.2258	32.3297	6.8745	0.3772
37	0.9414	5.9626	32.8706	7.7799	0.2778
38	0.9230	6.6405	33.828	6.6914	0.3319
39	0.9054	7.9345	34.8013	5.2862	0.4009
40	0.9408	11.3822	35.2263	3.6038	0.4265
41	0.9718	15.0539	34.8809	2.9153	0.3471
42	0.9824	18.2361	34.4149	2.5298	0.2849

ABR	GOF	coefficients			
color	adj. R^2	a_i	b_i	c_i	d_i
1	0.9768	9.4161	31.8372	5.1944	0.2079
2	0.9679	8.9927	31.3391	5.3012	0.2422
3	0.9781	9.0854	31.9894	5.6301	0.1459
4	0.9682	9.7410	32.9077	4.9153	0.2365
5	0.9682	10.0699	33.5090	4.9759	0.1748
6	0.9634	10.5713	33.4695	4.3783	0.2807
7	0.9749	11.7730	33.2090	3.9545	0.2732
8	0.9729	11.8048	33.0248	3.7414	0.3393
9	0.9759	12.2417	32.3127	3.6868	0.3126
10	0.9636	11.1394	32.2255	4.1219	0.2909
11	0.9244	9.9389	31.9093	4.1282	0.4262
12	0.9355	7.2652	32.4887	5.9088	0.3736
13	0.9313	6.3590	33.0081	7.4124	0.2571
14	0.9396	6.0825	33.7573	7.6819	0.2685
15	0.9429	5.6471	34.7604	8.3479	0.2569
16	0.9679	6.0105	34.6211	7.3194	0.3441
17	0.9389	6.1434	34.6808	7.1892	0.3393
18	0.9489	6.5330	33.9145	6.4573	0.3942
19	0.9263	7.2384	33.8770	5.3898	0.4820
20	0.9550	7.8194	33.0913	5.3805	0.3973
21	0.9742	7.7103	31.9129	5.1316	0.4667
22	0.9554	7.7273	31.2075	5.2751	0.4336
23	0.9380	7.4175	30.2305	5.6554	0.4008
24	0.9554	6.7559	29.7324	5.4401	0.5446
25	0.9258	5.4589	30.2321	7.0195	0.5013
26	0.8842	4.4785	31.4263	9.4882	0.3857
27	0.8850	4.3617	33.1516	10.3863	0.3079
28	0.8519	4.9499	35.3915	9.1483	0.3084
29	0.8806	5.7780	36.3703	5.8448	0.6272
30	0.9293	7.3518	36.1880	4.9598	0.5527
31	0.9566	7.7111	35.8412	4.3158	0.6408
32	0.9624	8.1670	34.9808	4.5050	0.5435
33	0.9672	8.4651	34.5726	4.5209	0.5026
34	0.9719	8.6853	34.0493	4.7860	0.4113
35	0.9654	8.2181	33.4321	4.9449	0.4371
36	0.9749	8.6030	33.0267	4.6504	0.4545
37	0.9693	8.3520	32.7635	5.1253	0.3770
38	0.9655	7.5223	32.1493	5.6623	0.3829
39	0.9379	6.4382	32.5080	6.7188	0.3645
40	0.9468	6.2240	32.3134	6.8049	0.3895
41	0.9715	6.0901	32.4849	7.3403	0.3245
42	0.9711	5.9673	33.5075	7.5549	0.3140

43	0.9920	20.7631	33.6742	2.3285	0.2235		43	0.9598	5.7631	33.2689	7.7339	0.3281
44	0.9920	19.4501	32.7112	2.4711	0.2314		44	0.9568	5.5256	32.3732	8.0150	0.3360
45	0.9586	14.9228	32.1784	2.7002	0.4465		45	0.9628	5.4290	32.4576	7.9843	0.3620
46	0.8514	9.5939	31.9281	3.2104	0.7095		46	0.9076	5.0328	32.3610	8.6369	0.3587
47	0.8673	5.6170	34.2469	8.2197	0.2838		47	0.9007	5.6421	33.3450	7.4638	0.3962
48	0.9535	5.7514	34.5855	8.1935	0.2574		48	0.9095	6.2822	34.2144	6.0321	0.5130
49	0.9471	5.8867	34.0122	7.9776	0.2619		49	0.9227	6.8444	34.3177	5.5003	0.5199
50	0.9717	6.3316	34.7587	7.3595	0.272		50	0.9011	7.7838	34.5148	4.6485	0.5604
51	0.9631	6.4333	34.5689	7.0431	0.3076		51	0.9606	7.9737	33.8098	5.0340	0.4509
52	0.9549	6.8914	34.244	6.3486	0.3508		52	0.9642	8.0626	33.7038	5.1495	0.4127
53	0.9691	7.7868	33.9974	5.5295	0.3701		53	0.9753	9.4273	33.2928	4.6987	0.3357
54	0.9690	8.0479	33.4471	5.3175	0.3773		54	0.9340	8.6288	32.7119	5.1326	0.3360
55	0.9697	8.0828	33.424	5.0505	0.432		55	0.9680	8.1461	33.2196	5.8156	0.2505
56	0.9792	8.6937	33.0479	4.9067	0.3811		56	0.9773	7.7832	33.2780	6.0728	0.2535
57	0.9884	8.5415	33.0539	4.9535	0.3907		57	0.9783	7.5129	33.5927	6.1130	0.2906
58	0.9821	8.5362	32.995	5.0298	0.3734		58	0.9738	8.2189	33.6096	5.9163	0.2158
59	0.9811	8.1938	33.0944	5.4584	0.3239		59	0.9749	7.5707	33.6519	6.1760	0.2676
60	0.9889	7.9266	33.2915	5.8184	0.2852		60	0.9717	8.4149	33.3366	5.7966	0.2116
61	0.9860	7.8825	33.7638	5.8570	0.2839		61	0.9883	9.0621	33.3384	5.4897	0.1847
62	0.9736	9.0980	34.1577	5.1566	0.2632		62	0.9924	9.0497	33.3277	5.3775	0.2148
63	0.9748	10.0891	34.6557	4.7225	0.2430		63	0.9825	9.4639	32.8530	5.0644	0.2351
64	0.9853	12.216	34.5252	3.683	0.3165		64	0.9653	9.3738	32.7069	5.3189	0.1817

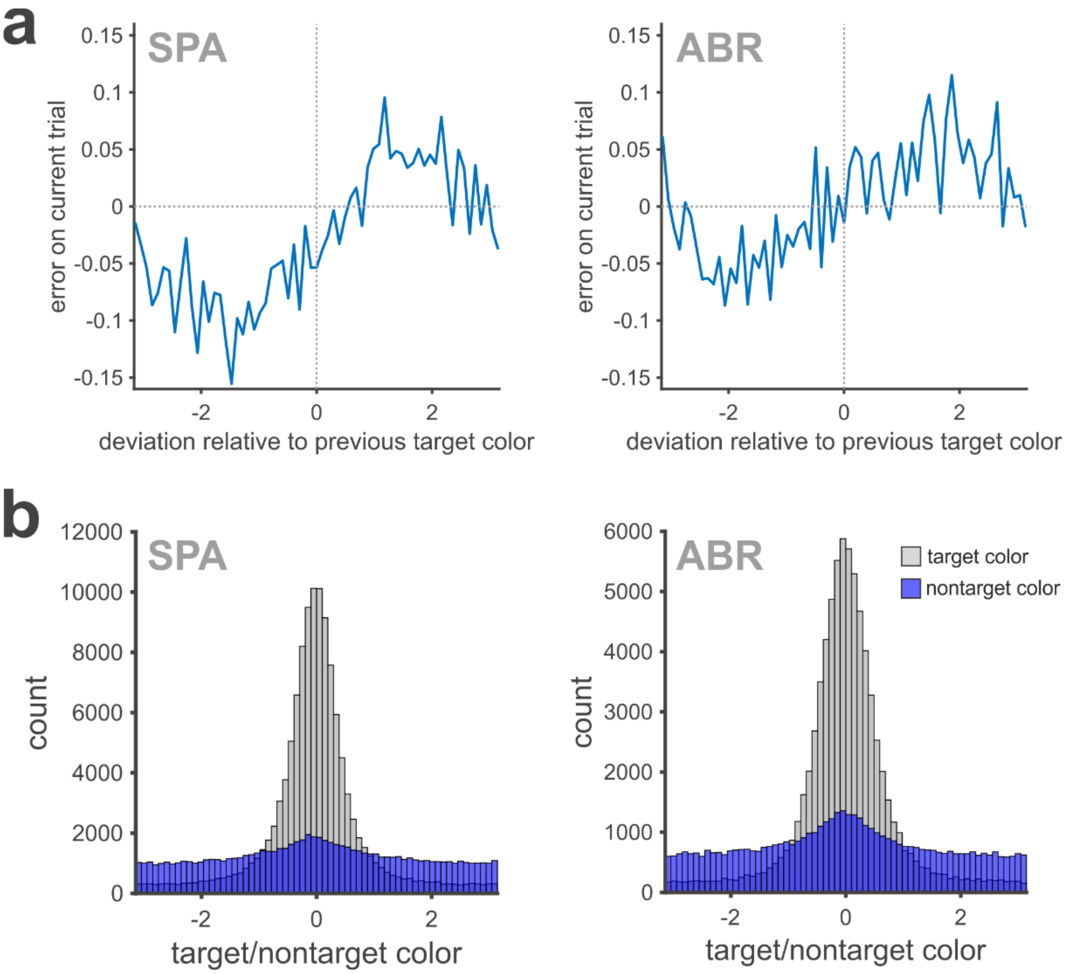

Fig. S5: Two additional factors, the previous target color and nontarget color in load 2 trials, affected the response behavior. a Serial bias in delayed estimation. Responses of both birds were shifted in direction of the previous target color in 52% (SPA) and 51% (ABR) of trials. The direction of the current response bias (i.e., error on the current trial) was dependent on the deviation of the current target color relative to the previously presented color. Both showed the same direction within the color wheel, i.e., the responses of both birds were attracted to the previous target color. This effect was also dependent on the color deviation magnitude. Deviations around 1.5 radians resulted in the strongest serial biases. **b** Response distributions aligned to target and nontarget color (pooled across all trials and target or nontarget colors). Histograms show angular deviation of responses aligned to target and nontarget color for all completed trials (in radian, -pi to pi). In a small proportion of trials, the birds appeared to report the nontarget color instead of the target color (visible in the small peak of the nontarget response distribution).

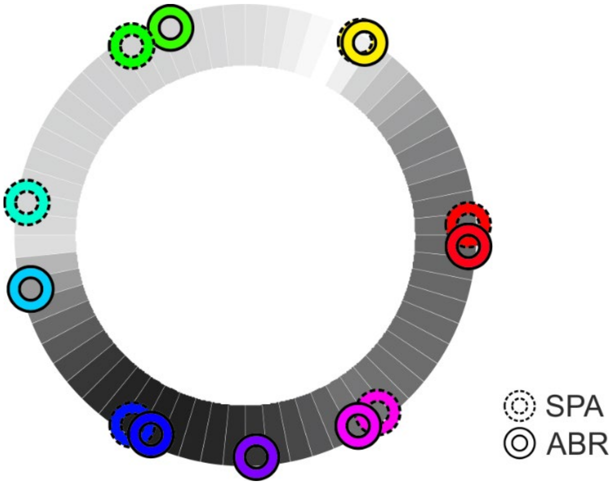

Fig. S6: Differences in luminance cannot solely explain position of all identified attractor colors. Attractor positions are visualized with respect to stimulus luminance and color-coded per bird (SPA: dashed, ABR: solid). Grey visualization of the color wheel choice stimulus symbolizes normalized luminance values per sample color (dark colors represent lower luminance values). Although the positions of some attractors align with peaks (yellow) or troughs (dark blue) in luminance, not all attractor states can be explained solely based on luminance.

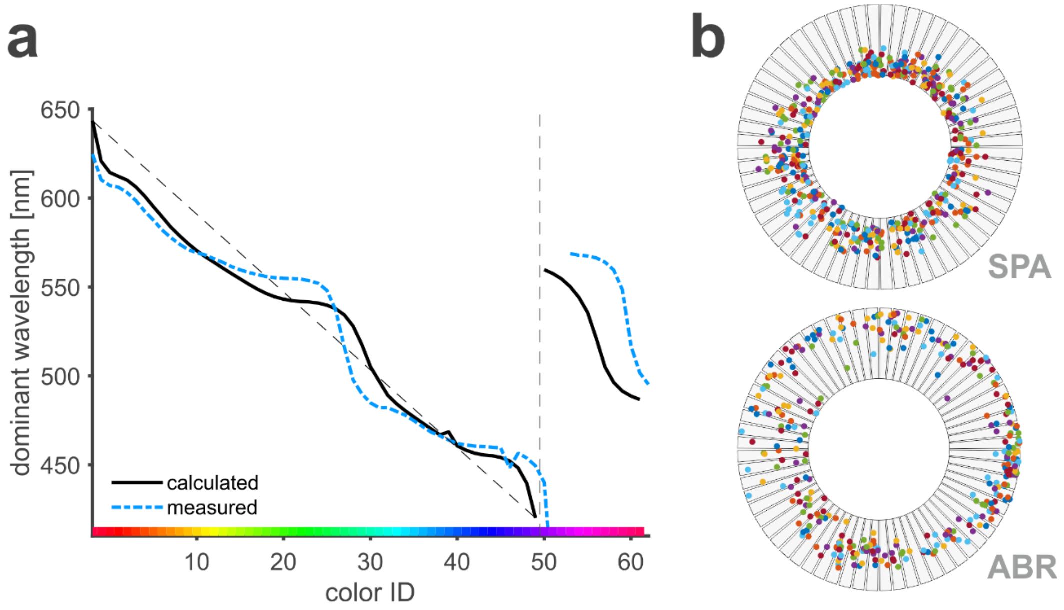

Fig. S7: Additional methodological details of color stimuli and response behavior. a Post-hoc measured dominant (and complementary) wavelengths of all colors used in the experiment. Colors were defined as equidistant hues in the HSV color space (hue, saturation, value) and then converted into RGB values for display. Shown are dominant wavelengths calculated based on the HSV values per sample color (black curve) and measured on the experimental monitor (blue dashed curve). All values deviate from a uniform distribution of dominant wavelengths in a somewhat comparable fashion. Vertical grey dashed line differentiates colors with a dominant wavelength (left) from colors with only a complementary wavelength (right). **b** Distribution of all pecks made to the color wheel choice stimulus during one exemplary experimental session per bird. Both birds showed quite even peck distributions throughout individual sessions. SPA tended to respond mostly to inner sections, ABR pecks were mostly within the outer sections of the color wheel. The birds showed no apparent spatial bias. Nevertheless, the color wheel was randomly rotated on each trial to prevent potential location biases (colors are not related to sample color per trial and solely represent individual pecks).

Table S5: Measured and calculated dominant wavelengths of all 64 colors used in the delayed estimation experiment. Colors within the purple range are described by a complementary instead of dominant wavelength (refers to color ID 53 – 62). Luminance values of all sample stimuli are visualized in Fig S6.

color ID	dominant wavelengths		RGB values		
	calculated	measured	R	G	B
1	612.1	607	1	0	0
2	610	606	1	0.0938	0
3	606.2	603	1	0.1875	0
4	601.1	598.5	1	0.2813	0
5	595.2	592.6	1	0.3750	0
6	589.5	587.6	1	0.4688	0
7	583.7	582.1	1	0.5625	0
8	578.9	577.8	1	0.6563	0
9	574.4	574.1	1	0.7500	0
10	570.4	571	1	0.8438	0
11	566.9	569.2	1	0.9375	0
12	563.8	567.9	0.9688	1	0
13	560.6	565.8	0.8750	1	0
14	557.5	563.8	0.7813	1	0
15	554.5	561.4	0.6875	1	0
16	551.5	559.6	0.5938	1	0
17	548.7	558.1	0.5000	1	0
18	546.3	556.9	0.4063	1	0
19	544.4	555.8	0.3125	1	0
20	543.1	555.2	0.2188	1	0
21	542.2	554.8	0.1250	1	0
22	541.8	554.7	0.0313	1	0
23	541.5	554.4	0	1	0.0625
24	540.9	553.4	0	1	0.1563
25	539.7	552	0	1	0.2500
26	537.7	548	0	1	0.3438
27	534.2	536.8	0	1	0.4375
28	528	516	0	1	0.5313
29	517.7	497.7	0	1	0.6250
30	505.2	490	0	1	0.7188
31	497	484.8	0	1	0.8125
32	489	482.1	0	1	0.9063
33	484.3	482.1	0	1	1
34	480.4	480	0	0.9063	1
35	476.9	477.9	0	0.8125	1
36	473.5	474.8	0	0.7188	1
37	470.1	471.5	0	0.6250	1
38	466.8	468.3	0	0.5313	1
39	468.5	466.3	0	0.4375	1
40	460.6	463.9	0	0.3438	1
41	458.2	462	0	0.2500	1
42	456.5	461.1	0	0.1563	1
43	455.5	460.5	0	0.0625	1
44	455.1	460.2	0.0313	0	1
45	454.1	459.9	0.1250	0	1
46	452.2	459.4	0.2188	0	1
47	448.2	448.3	0.3125	0	1
48	439.5	456.5	0.4063	0	1

49	420.1	453.8
50	559.7	449.4
52	557.5	440.1
52	554.5	379.2
53	550.1	568.7
54	544.1	567.8
55	535.6	567.4
56	521	566.3
57	505	563.9
58	496.3	559.5
59	491.4	548.4
60	488.6	516.7
61	486.7	501.7
62	643.4	495.2
63	620.7	624.7
64	614.3	610.2

0.5000	0	1
0.5938	0	1
0.6875	0	1
0.7813	0	1
0.8750	0	1
0.9688	0	1
1	0	0.9375
1	0	0.8438
1	0	0.7500
1	0	0.6563
1	0	0.5625
1	0	0.4688
1	0	0.3750
1	0	0.2813
1	0	0.1875
1	0	0.0938

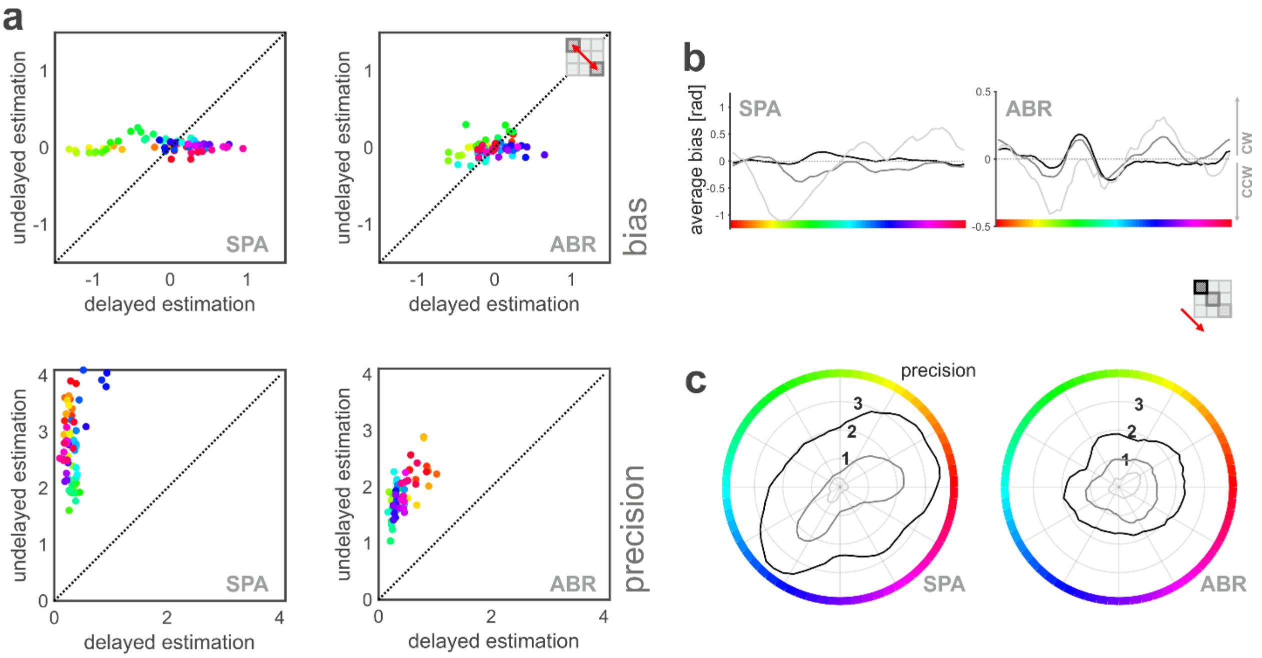

Fig. S8: Differences in bias and precision per target color for undelayed and delayed estimation. a Colored dots represent the comparison of bias (top) and precision (bottom) between trial type with lowest vs. highest WM demands (i.e., 1 vs. 9). **Bias** indicates the average angular deviation in radian between target color and reported color (positive = CW, negative = CCW shifts). Differences in response bias were more pronounced in delayed estimation trials for both birds, particularly SPA. Average bias was more spread along the delayed estimation (mean absolute bias across all colors, delayed: 0.4525 ± 0.0433 vs. undelayed: 0.0591 ± 0.0068), indicating more pronounced memory effects for SPA (top, left). The bias pattern of ABR was less clear, suggesting a mix of mnemonic (delayed: 0.1956 ± 0.0194) and perceptual (undelayed: 0.0765 ± 0.0092) causes (top, right). **Precision** was calculated as inverse of the circular standard deviation per target color. Response precision was clearly higher in the undelayed estimation for all target colors in both birds (SPA, delayed: 0.3216 ± 0.0196, undelayed: 2.7479 ± 0.0813; ABR: delayed 0.4218 ± 0.0252, undelayed: 1.8661 ± 0.0461).

Response bias and precision were modulated by memory demands. b Average response bias per target color increased with memory demands. Both birds showed a successive increase in bias strength with increasing memory demands. Bias was calculated along the diagonal (per target color and trial type, then calculated in a sliding window of ±3 mirroring the full reward range). **c** Response precision was color-dependent and decreased with increasing memory demands. Precision was calculated as inverse of circular standard deviation per target color and trial type, then calculated in a sliding window of ±3 mirroring the full reward range. Color bar/ring show all 64 target colors, line contrast visualizes WM demand (black: load1 + ‘simultaneous’/undelayed; dark grey: load2 sim cue + SD; light grey: load2 retro cue + LD). Response bias (circular mean error) and precision (inverse of circular standard deviation, corrected for chance) were calculated using code provided by Bays et al.[1] (http://bayslab.com).

Supplementary references

1. Bays, P. M., Catalao, R. F. G. & Husain, M. The precision of visual working memory is set by allocation of a shared resource. *Journal of Vision* **9**, 7 (2009).
2. Hardman, K. O., Vergauwe, E. & Ricker, T. J. Categorical working memory representations are used in delayed estimation of continuous colors. *Journal of Experimental Psychology: Human Perception and Performance* **43**, 30–54 (2017).
3. Souza, A. S., Overkott, C. & Matyja, M. Categorical distinctiveness constrains the labeling benefit in visual working memory. *Journal of Memory and Language* **119**, 104242 (2021).
