# Supplementary material for: Corvids optimize working memory by categorizing continuous stimuli
Source: Commun Biol. 2023 Nov 6;6:1122. doi: 10.1038/s42003-023-05442-5 (PMC10628182; doi:10.1038/s42003-023-05442-5)
Supplement: Supplementary file 2 — Description of Additional Supplementary Files [file 42003_2023_5442_MOESM2_ESM.pdf]

Description of Additional Supplementary Files

File name: Supplementary Data 1
Description: Numerical source data for graphs and charts
